# Bone Regeneration in Rat Calvaria Using 3D-Printed Scaffolds with Graded Porosity and In Vitro Degradation

**DOI:** 10.1021/acsomega.5c06247

**Published:** 2025-09-17

**Authors:** Lucía Pérez-Sánchez, Mariana Nataly Carbajal-Casique, Rafael Álvarez-Chimal, Marco A. Alvarez-Perez, Juan José Montesinos, Monserrat Llaguno-Munive, Janeth Serrano-Bello

**Affiliations:** † Laboratorio de Bioingeniería de Tejidos, División de Estudios de Posgrado e Investigación, Facultad de Odontología, Universidad Nacional Autónoma de México, 318340 Circuito Exterior s/n. Cd. Universitaria, Mexico City 04510, México; ‡ Mesenchymal Stem Cells Laboratory, Oncology Research Unit, Oncology Hospital, National Medical Center (IMSS), Mexico City 06720, Mexico; § Laboratorio de Física Médica, Subdirección de Investigación Básica, 42597 Instituto Nacional de Cancerología, Mexico City 14080, México

## Abstract

Craniofacial bone defects present a significant clinical challenge due to their structural complexity and potential neurological implications. In this study, a three-dimensional (3D) polylactic acid (PLA) scaffold with graded porosity and three pore types was fabricated and subjected to a controlled in vitro degradation process. Dental pulp stem cells (DPSCs), which are known for their osteogenic potential, were seeded on the scaffolds to evaluate their osteoconductive performance in a critical-size calvarial defect model in Wistar rats. In vitro assays revealed no significant changes in surface morphology, weight, pH, and mechanical properties over 0, 60, 100, 140, and 180 days of degradation. However, scaffolds degraded for 60 days demonstrated enhanced biological activity in cell-based assays and were therefore selected for in vivo implantation. Microcomputed tomography and bone mineral density analysis indicated that the group receiving degraded scaffolds without cells exhibited the most substantial new bone formation, suggesting effective osteoconductive properties. These findings represent a promising step toward translational medicine and highlight the potential for clinical application, pending further preclinical validation.

## Graphical abstract

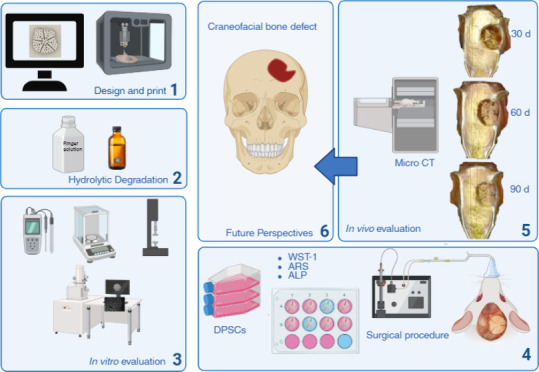

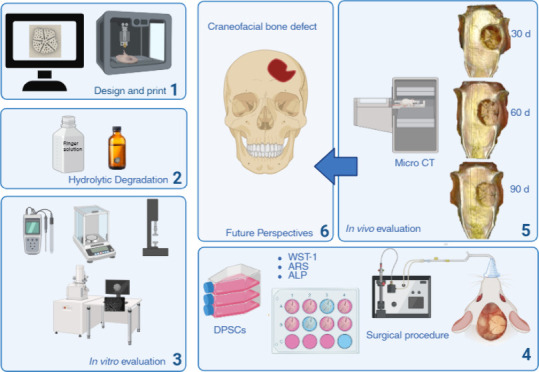

## 1 Introduction

Bone is the second most transplanted tissue globally;^1^ however, its regeneration remains a clinical challenge due to the structural and functional complexity of its extracellular matrix, which is hierarchically organized and composed of both organic and inorganic components.^2,3^ This challenge is further amplified in anatomically complex regions such as the craniofacial area, where bone defects resulting from trauma, congenital malformations, or tumor and cystic pathologies often exceed the critical threshold for self-regeneration.^4^ These critical-sized defects do not heal spontaneously and require surgical intervention,^5^ as they can compromise both aesthetic and neurological functions.^6–8^

In recent years, craniofacial tissue engineering has incorporated emerging technologies such as three-dimensional bioprinting, smart scaffolds fabricated from innovative materials, and bioactive materials with controlled release capabilities. These advances have expanded the potential for personalized and adaptive bone regeneration.^9–12^ Nevertheless, many of these strategies still face technical and translational challenges, prompting continued exploration of customizable scaffold designs using accessible technologies such as 3D printing.

Despite growing technological development, clinical studies evaluating 3D-printed scaffolds for bone regeneration remain limited and often involve a small number of patients, which restricts the generalizability of their findings. In this context, it is essential to continue strengthening preclinical research to enable the safe and effective translation of these approaches into broader clinical applications.^13–15^

On the other hand, the current standard treatment for bone defects involves grafting, with autologous bone grafts considered the clinical gold standard due to their inherent osteogenic potential. However, these grafts present limitations, including donor site morbidity and limited availability of harvestable tissue.^16,17^ In this context, tissue engineering has gained relevance by developing three-dimensional (3D) scaffolds designed to mimic the structural and functional characteristics of the extracellular matrix, providing mechanical support and guiding tissue regeneration.^18,19^

Advances in 3D printing technologies, particularly fused deposition modeling (FDM), have enabled precise customization of scaffold geometry, pore size, and porosity using medical imaging data.^20,21^ Among the materials employed, polylactic acid (PLA) stands out due to its biocompatibility and commercial availability. However, it also presents certain limitations, including a slow degradation rate and mechanical properties inferior to those of cortical bone.^22,23^ To address these issues, strategies such as controlled hydrolysis have been explored to accelerate its degradation and enhance in vivo performance without compromising structural integrity.^24^

Beyond structural design and degradation control, mesenchymal stem cells (MSCs) are another key tissue engineering element. These cells are valued for their self-renewal capacity, immunomodulatory properties, and osteogenic differentiation potential.^25–28^ Although bone marrow-derived MSCs (BMSCs) are widely used, their clinical application is limited by the invasive nature of the harvesting procedure and the age-related decline in their regenerative capacity.^25–32^

As an alternative, MSCs derived from oral tissues,^33–35^ particularly dental pulp stem cells (DPSCs), have been proposed. DPSCs offer several advantages, including minimally invasive collection, osteogenic differentiation potential, and their neural crest origin, which makes them especially suitable for applications in the craniofacial complex. Several studies have shown that DPSCs can produce more mineralized matrix than BMSCs and exhibit a similar gene expression profile, supporting their use in bone regeneration strategies.^36–42^

The present study aimed to evaluate bone regeneration in critical-sized calvarial defects in Wistar rats using customized 3D-printed scaffolds with graded porosity, fabricated via FDM using PLA. Notably, this type of scaffold has not been previously reported. A degradation study was conducted, and DPSCs were seeded onto the scaffolds to assess their osteogenic differentiation potential in vitro. Finally, the scaffolds were implanted into critical-sized calvarial defects in Wistar rats to analyze their in vivo osteoconductive capacity. Microcomputed tomography was used to evaluate the outcomes and determine the scaffold’s effectiveness in promoting new bone formation.

## Materials and Methods

### 2.1 Scaffold Design

The scaffold was designed to match a 9 mm diameter and 1 mm thick critical calvarial defect in rats, incorporating a graded porosity architecture with three distinct pore types. The design process followed the methodology previously established by our group [Pérez et al.].^43^ A DICOM file obtained from micro-CT imaging using the ALBIRA ARS scanner (ONCOVISION, INCan) was converted into a 3D STL model using InVesalius 3.1. The scaffold geometry was then refined using 3D Builder, Meshmixer, and Rhinoceros 7 to accurately reproduce the defect anatomy and implement the targeted internal porosity. The final STL model was converted to g-code using Ultimaker Cura 5.6.0 for 3D printing.

### 2.2 Scaffold Printing

Scaffolds were fabricated using the FDM process with a MONOPRICE SELECT MINI 3D printer and a 1.75 mm white PLA filament (3D-Market). The extrusion temperature was set at 205 °C, with a build plate temperature of 50 °C.

To ensure structural consistency, the printing parameters used were standardized and validated in our previous study [Pérez et al.].^43^ Therefore, the specific settings are not repeated here. A total of 250 scaffolds were printed under optimized conditions.

### 2.3 In Vitro Hydrolytic Degradation of the PLA Scaffolds

The in vitro degradation experiment was conducted by placing individual 3D-printed scaffolds into amber glass containers, each filled with 134 mL of Ringer’s lactate solution. The solution volume was determined based on the protocol described by Felfel et al.^44^ The composition per 100 mL included sodium chloride (0.600 g), potassium chloride (0.030 g), calcium chloride dihydrate (0.020 g), and sodium lactate (0.310 g).

The scaffolds were evaluated on days 0, 60, 100, 140, and 180. Ten scaffolds were used on each evaluation (*n* = 50 in total), and all samples were incubated at 37 °C in a laboratory oven (FELISA brand) to simulate physiological conditions.

### 2.4 Surface Morphology and Scaffold Porosity Evaluation

Morphological analysis was conducted by scanning electron microscopy (SEM) on a JEOL 5600LV system operated at 25 kV with 25× magnification, employing both secondary and backscattered electrons. Five scaffolds were analyzed at each in vitro degradation time point (0, 60, 100, 140, and 180 d; n = 25). Prior to imaging, all the samples were coated with a conductive gold layer using plasma-assisted sputtering. The SEM analysis was performed at the Central Microscopy Laboratory of IF-UNAM.

The pore size was assessed in a single cross-sectional evaluation of the scaffolds, measuring pores from the center toward the periphery. Because no significant surface changes were observed over time, comparisons across the degradation intervals were not performed. A total of 130 pores were measured using the ImageJ software (version IJ-1.54-win-java8).

The porosity was determined using a gravimetric method based on Archimedes’ principle, with 100 mL of deionized water as the displacement medium.^45^ Measurements were performed in triplicate for each degradation time point (*n* = 15). Porosity (%) was calculated using the following equation:

Porosity=(Wsat−Wdry)/(Wsat−Wsus)x100%

Where:

Wsat: weight of the water-saturated scaffold

Wdry: dry weight of the scaffold

Wsus: weight of the scaffold suspended in water

### 2.5 Measurement of pH and Scaffold Weight

To monitor physicochemical changes associated with in vitro degradation, the pH of the Ringer’s lactate solution (PiSA) in which the scaffolds were immersed was measured on each evaluation day (0, 60, 100, 140, and 180 days). Eight scaffolds were analyzed on each evaluation day (*n* = 40). Following the manufacturer’s instructions, a calibrated digital pH meter (PEAKINSTRUMENTS) was used for all measurements.

After removal from the solution, the scaffolds were dried in an incubator (FELISA) at 37 °C for 30 min and weighed using an analytical balance (aeADAM) to assess the potential mass loss due to degradation.

### 2.6 Mechanical Testing

Uniaxial compression tests were performed using a universal testing machine (INSTRON 5567) equipped with a 4.5 kN load cell at a 1 mm/min crosshead speed under standard environmental conditions (23 °C and 50 % relative humidity). Five scaffolds were tested on each evaluation day (0, 60, 100, 140, and 180 d; *n* = 5 per group).

The resulting stress–strain curves calculated each specimen’s elastic (Young’s) modulus and maximum compressive strength.

### 2.7 Cell Culture

Mesenchymal stem cells derived from dental pulp (DPSCs), previously characterized at the Medical Research Unit for Oncologic Diseases, Oncology Hospital, Centro Médico Nacional Siglo XXI, IMSS,^46^ were cultured and expanded in Dulbecco’s Modified Eagle’s Medium (DMEM, GIBCO) supplemented with 10 % fetal bovine serum (FBS, GIBCO) and an antibiotic solution containing penicillin (100 U/mL), streptomycin (100 μg/mL), and amphotericin B (0.3 μg/mL).

The cell cultures were maintained at 37 °C in a humidified incubator under 95 % air and 5 % CO_2_. Before cell seeding, the 3D-printed scaffolds were sterilized using ethylene oxide gas. DPSCs were seeded onto scaffolds at a density of 2 × 10^4^ cells/mL at each degradation time point (0, 60, 100, 140, and 180 d) in triplicate using 46-well culture plates.

Every third day, the medium was replaced with fresh osteogenic induction medium containing 50 μM ascorbic acid, 10 mM β-glycerophosphate, and 10^–7^ M dexamethasone, as described by Phillips et al.^47^

### 2.8 Cell Viability Assay (WST-1)

The viability of DPSCs seeded on the 3D-printed scaffolds was assessed using the Cell Proliferation Reagent WST-1 assay (SIGMA-Aldrich). On days 1, 7, and 14, the cultures were incubated with 20 μL WST-1 reagent in 200 μL culture medium for 4 h. After incubation, 100 μL of the supernatant was transferred to a new plate, and the absorbance was measured at 450 nm using a microplate reader (CHROMATE, AWARENESS TECHNOLOGY). The assay was performed in triplicate for each scaffold degradation group (0, 60, 100, 140, and 180 days).

### 2.9 Alkaline Phosphatase (ALP) Assay

To evaluate ALP activity in DPSCs seeded scaffolds cultured in osteogenic medium and categorized according to their degradation time (0, 60, 100, 140, and 180 days), a colorimetric ALP assay kit (ABCAM, Cambridge, UK) was used. This assay is based on the dephosphorylation of p-nitrophenyl phosphate (pNPP) by ALP, forming a yellow-colored product.

After incubation, the 3D scaffolds were rinsed with PBS and transferred to 1.5 mL Eppendorf tubes. Cells were lysed using a 0.1% Triton X-100 solution (ALP assay buffer) for 30 min. From the resulting lysate, 25 μL aliquots were taken in triplicate and placed into the wells of a 96-well plate. Next, 50 μL of pNPP substrate solution (5 mM) was added to each well and incubated for 30 min. The reaction was stopped by adding 20 μL of stop solution (NaOH), and the absorbance was measured at 405 nm using an ELISA plate reader (CHROMATE, AWARENESS TECHNOLOGY). ALP activity was calculated as the amount of pNPP generated per unit sample volume per minute of the reaction. The assay was performed on days 7 and 14 of culture.

### 2.10 Alizarin Red Staining (ARS) Assay

The alizarin red staining (ARS) assay was used for the visual detection of calcium deposits formed by DPSCs cultured in osteogenic medium on 3D-printed scaffolds classified by degradation time. Staining was performed using commercial ARS solution (40 mM, pH 4.2; Osteogenesis Kit, Millipore). The scaffolds were gently rinsed with PBS and fixed with 4% paraformaldehyde for 24 h, followed by three washes with distilled water.

After fixation, the scaffolds were stained with the ARS solution for 30 min. Excess stain was removed with multiple distilled water washes, and images were acquired using a stereomicroscope (OLYMPUS DSX-HRSU, Tokyo, Japan).

The stained scaffolds were transferred to 1.5 mL Eppendorf tubes for quantification, and 200 μL of 10% acetic acid was added. The samples were agitated for 30 min, incubated at 85 °C for 10 min, and placed on ice for 5 min. The tubes were then centrifuged at 13,000 rpm for 15 min. Then, 75 μL of 10% ammonium hydroxide was added to neutralize the solution. Finally, 200 μL of the supernatant was transferred to a 96-well plate, and the absorbance was measured at 405 nm using a plate reader. The assay was performed on days 7 and 14 of culture.

### 2.11 General Surgical Procedure in Wistar Rats

Twenty male Wistar rats (18 weeks old, approximately 250 g) were randomly assigned to four experimental groups (*n* = 5 per group):

1) Control (scaffold with graded porosity).
2) Scaffolds seeded with DPSCs induced toward osteogenic lineage.
3) Scaffolds were subjected to *in vitro* degradation and seeded with DPSCs.
4) Scaffolds subjected to *in vitro* degradation without cells.

All surgical procedures were conducted under the institutional animal care guidelines approved by the Internal Committee for the Care and Use of Laboratory Animals (CICUAL), under protocol number FO-M001–0009–2021, following the Mexican Official Standard NOM-062-ZOO-1999.

The animals were anesthetized via intramuscular injection of ketamine (80 mg/kg) and sedated with inhaled isoflurane (SOFLORAN VET) using a SomnoSuite Low-Flow Anesthesia System (Kent Scientific Corporation). The surgical site was shaved and disinfected with povidone-iodine. Local anesthesia was administered with mepivacaine HCl and epinephrine (DENTOCAIN, ZEICO).

A 3 cm linear incision was made through the skin and the periosteum of the calvaria to expose the cranial vertex. A critical-sized defect (9 mm diameter) was created using a trephine mounted on a surgical motor implant operating at 4000 rpm. The defect was positioned lateral to the sagittal suture on the frontal bone and included a portion of the temporal bone. Sterile phosphate-buffered saline (PBS) was used for irrigation throughout the procedure.

The bone fragments were carefully removed using a chisel to avoid damage to the dura mater. The surgical site was then rinsed with sterile PBS. The 3D-printed PLA scaffolds were implanted into the defects according to the classification of the previously listed groups.

Wound closure was achieved with continuous sutures using 4–0 polyglycolic acid. Postoperative clinical monitoring included evaluation of the general condition, pain assessment using the Rat Grimace Scale,^48^ and wound inspection for bleeding, discharge, swelling, or biomaterial extrusion.

Animals were housed under a 12-h light/dark cycle, maintained at 50% relative humidity, and provided Rodent Diet 5001 ad libitum.

### 2.12 Evaluation of Implanted Scaffolds by Microcomputed Tomography (μCT)

To assess the bone mineral density (BMD) of the newly formed tissue, microcomputed tomography (μCT) scans were acquired 30, 60, and 90 days postimplantation of the 3D-printed PLA scaffolds. The field of view was centered on the calvarial region using a current of 0.4 mA, a voltage of 35 kV, and 1000 projections to obtain high-resolution images.

For BMD quantification, cubic volumes of interest (3 mm per side) were defined within the defect area and in regions of the native bone (control). Image analysis was performed using the PMOD software. BMD values were calibrated against a known hydroxyapatite standard to obtain concentrations in grams of hydroxyapatite per cubic centimeter (gHA/cc) for both the control and experimental samples.

Three-dimensional reconstructions of the samples were processed using OsiriX MD software, which enabled qualitative comparisons across all experimental groups.

### 2.13 Animal Euthanasia

At the end of the designated experimental period, the animals were euthanized with carbon monoxide to ensure a painless and humane procedure, leading to cardiorespiratory arrest. This protocol was conducted according to the Mexican Official Standard NOM-062-ZOO-1999. Subsequently, osteotomy was performed to remove the calvarial bone in the defect area. The procedure was carried out using a high-speed rotary instrument under continuous irrigation with running water, maintaining a 10 mm safety margin around the defect perimeter. The retrieved samples were gently rinsed and fixed in 10% neutral buffered formalin for 24 h.

### 2.14 Statistical Analysis

All samples were run in triplicate unless otherwise mentioned. The data was represented as mean ± standard deviation. The statistical significance of the difference was measured using one-way or two-way ANOVA with post hoc Tukey test (v9.4.1, Graphpad Prism). For porosity measurement, the Mann–Whitney U test was performed using SPSS software. The statistical threshold for significance was set at *p < 0.05.

## 3 Results

### 3.1 3D Scaffold Design and Printing

To design the 3D scaffold, a bone defect was created in the calvaria of Wistar rats as a framework, as shown in Figure 1(A). Subsequently, the slices obtained through micro-CT were processed in DICOM format using Osirix software and then converted into a 3D image in STL format using InVesalius software (Figure 1B). With the 3D image, the scaffold was designed with graded porosity and three types of pores using the Rhino 7 software, precisely adjusted to fit the size of the bone defect, as shown in Figure 1(C) and (D). Figure 1(D) on the left shows a front view of the bone defect with the 3D scaffold, whereas Figure 1(D) on the right presents the internal view of the bone defect with the scaffold. Once designed, the scaffold was printed using a Monoprice Select Mini FDM 3D printer, employing a white PLA polymer and following the printing parameters in the Ultimaker Cura software, as described in the methodology. The scaffold measured 9 mm in diameter and 1 mm in thickness, and the image clearly shows the porosity across the entire surface of the scaffold (Figure 1E).

**Figure 1:**
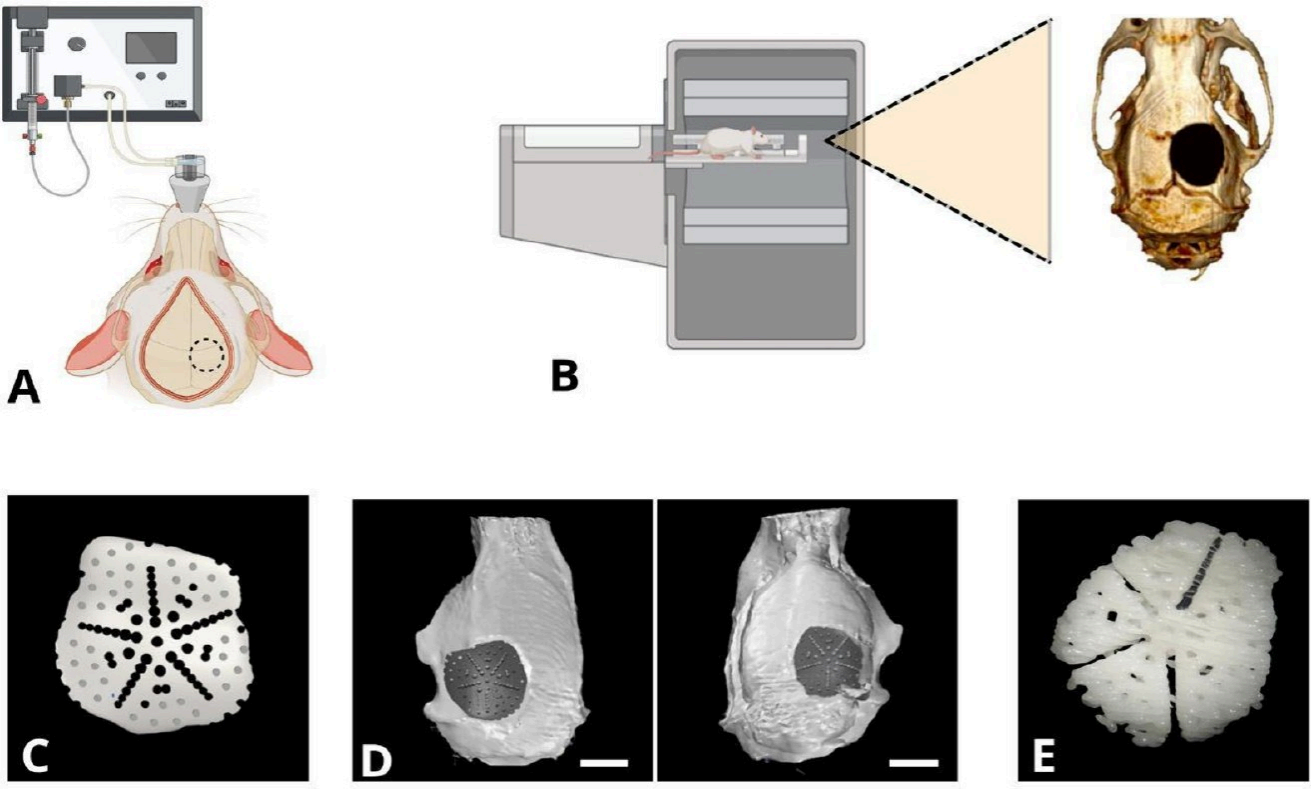
3D scaffold design and printing. (A) The bone defect created in the calvaria of a Wistar rat. (B) Image processing in DICOM format. (C) Scaffold design with graded porosity and three types of pores. (D) The adequate position of the scaffold is seen in the frontal and internal views of the bone defect (scale bar = 5 mm). (E) The printed PLA scaffold can be seen from a frontal view. Created using Biorender software and modified from Pérez et al.^43^

### 3.2 Pore Size Evaluation

Pore size analysis was performed using SEM micrographs and the ImageJ software. Measurements were taken radially from the center toward the edge of the scaffold, resulting in a histogram (Figure 2A) that reflected the graded porosity pattern. Larger pore sizes were concentrated in the center of the scaffold and gradually decreased toward the periphery. Figure 2A shows that the pore sizes ranged from a minimum of 200 μm to a maximum of 700 μm, with a mean pore size of 392.87 μm and the most frequent pore size being 300 μm. The highest frequency of pores fell within the lower-diameter range, with decreasing frequency as the diameter approached the upper range. Figure 2B shows a scaffold diagram illustrating the distribution of different pore sizes across the scaffold surface. The green color represents the pores between 200 and 300 μm. The orange color indicates pores between 450 and 500 μm. Finally, the blue color corresponds to pores ranging from 500 to 700 μm. Figure 2C displays the three types of pores: closed pores are marked with a dashed arrow, blind pores with a solid arrow, and open pores or microchannels are indicated by the arrowhead.

**Figure 2:**
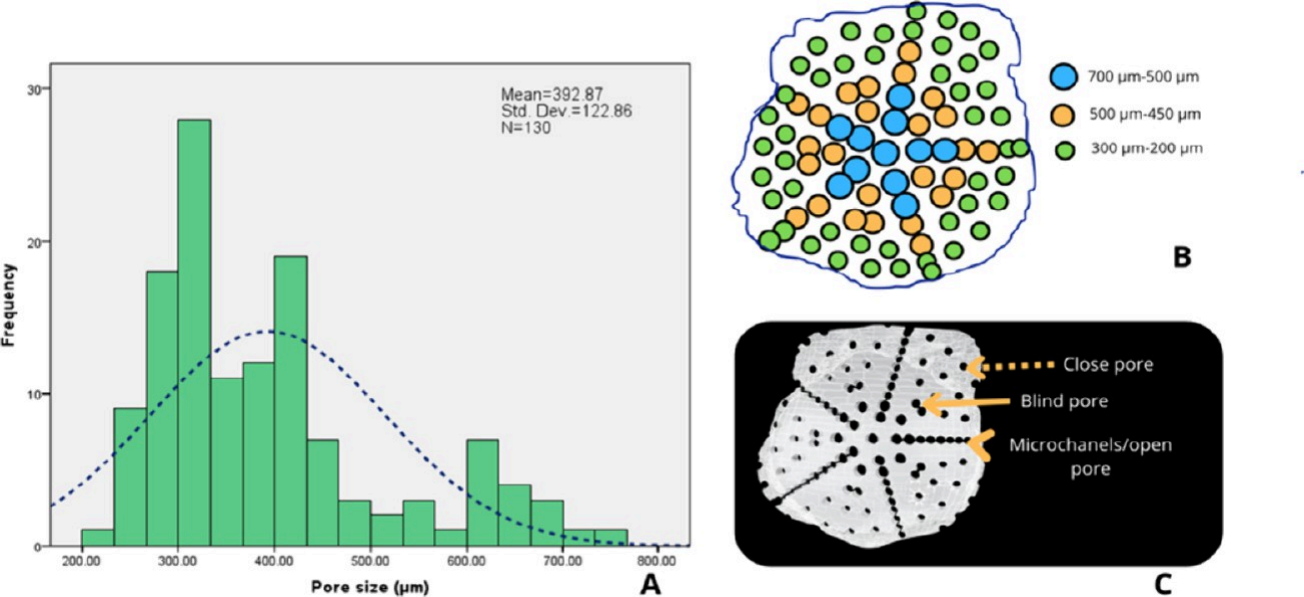
Graded porosity and three pore types. (A) Histogram showing a mean pore size of 392.87 μm ±  122.86, based on N = 130 pores. (B) Schematic representation of pore distribution across the scaffold surface: blue indicates pores ranging from 700 to 500 μm, orange from 500 to 450 μm, and green from 300 to 200 μm. (C) The diagram illustrates the three pore types, open, blind, and closed, as indicated by the arrows and modified from Pérez et al.^43^

### 3.3 Structural Changes in 3D Scaffolds after In Vitro Degradation

The surface structures of the constructs were evaluated after in vitro degradation for different periods (0, 60, 100, 140, and 180 days). Figure 3 shows the representative SEM images of the five regions of the scaffolds (columns) across various degradation time points (rows). This analysis aimed to identify potential surface modifications caused by the degradation process. Overall, no significant alterations were observed in the surface morphology compared with the original scaffold design.

**Figure 3:**
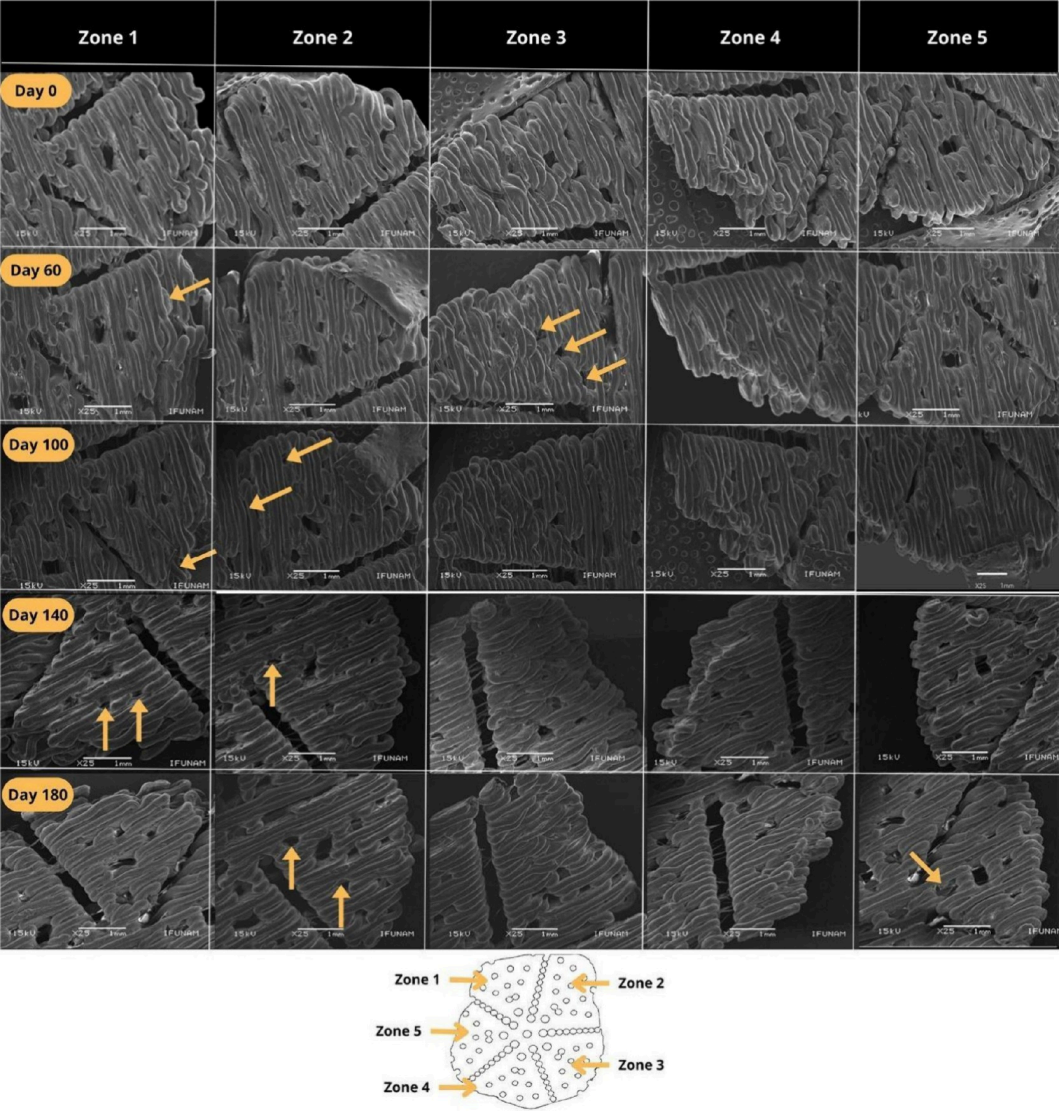
Scaffold surface after in vitro degradation at different time points (0, 60, 100, 140, and 180 days). Representative SEM images show five scaffold regions (columns) across various degradation time points (rows). On day 60, zone 1 displayed increased surface roughness (yellow arrow), whereas zones 3, 4, and 5 exhibited slight changes in pore shape. On day 100, zone 1 showed a filament with greater tortuosity, and zone 2 showed increased roughness. On day 140, zone 2 exhibited a limited change in the pore geometry. By day 180, the pores in zone 2 appeared to have slightly larger diameters.

In Figure 3, yellow arrows indicate some of the minor changes detected in the surface structure of the scaffolds relative to the control group (day 0, nondegraded). On day 60, in zone 1, the arrow highlights an area of increased surface roughness; zone 3 shows a slight change in pore shape, as seen in zones 4 and 5. On day 100, zone 1 showed a filament with greater tortuosity, and zone 2 showed a rougher surface texture. On day 140, zone 2 exhibited limited pore shape alteration, whereas on day 180, zone 2 exhibited pores that appeared to have slightly expanded diameters.

#### 3.3.1 Porosity Percentage Evaluation

The scaffold porosity was assessed as a general property to determine whether the degradation time impacted the porosity percentage. As shown in Figure 4A, there were no statistically significant differences between the groups. The average porosity was 64.5%.

**Figure 4:**
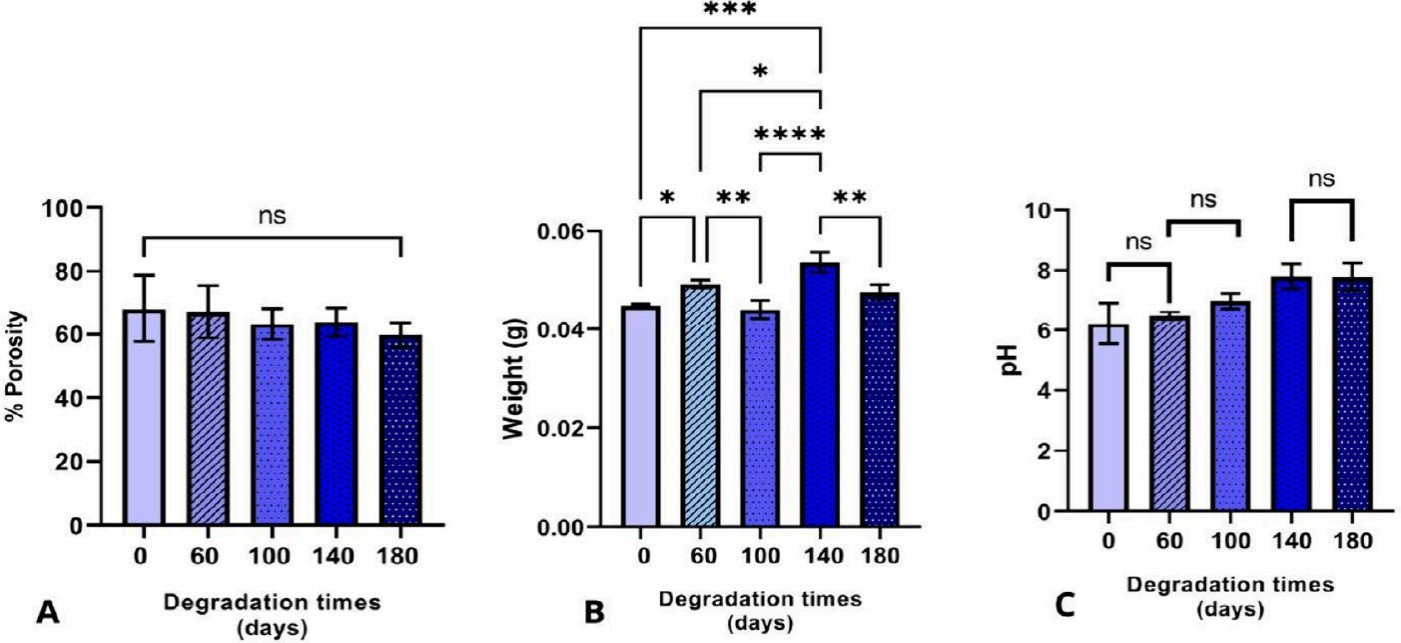
Evaluation of the porosity, weight, and pH. (A) Porosity percentage as a function of degradation time. No statistically significant differences were observed between the time points, with an average porosity of 64.5%. (B) Scaffold weights at the end of each degradation period. Statistically significant differences were found between day 0 vs days 60 and 140, day 60 vs days 100 and 140, day 100 vs day 140 and 180, and day 140 vs day 180. (C) pH of the Ringer’s lactate solution. The graph shows the progressive alkalinization of the solution during scaffold immersion over time, with statistically significant differences between groups indicated by asterisks: *p* < 0.05 (*), *p* < 0.01 (**), *p* < 0.001 (***), and *p* < 0.0001 (****).

#### 3.3.2 Measurement of Hydrogen Potential (pH) and Weight

The pH of the Ringer’s lactate solution in which the scaffolds were immersed was measured over time (Figure 4B). The results showed a general trend toward alkalinization with increasing degradation time, with significant differences observed between days 0 and 60 compared to days 140 and 180.

Scaffold porosity was evaluated as a general property to determine whether degradation time influenced the porosity percentage, as shown in Figure 4A. The results indicated no statistically significant differences in porosity between the groups, with an average value of 64.5%. The scaffold weight was also measured (Figure 4C) to assess whether degradation led to mass loss. A one-way ANOVA followed by Tukey’s post hoc test revealed statistically significant differences across several time points: day 0 vs days 60 and 140; day 60 vs days 100 and 140; day 100 vs day 140 and 180; and day 140 vs day 180, as illustrated in Figure 4.

### 3.4 Mechanical Testing

The mechanical properties of the scaffolds were evaluated and interpreted using stress–strain curves (Figure 5), which showed that the scaffolds exhibited high elastic deformability under low stress, with very similar curve profiles across groups.

**Figure 5:**
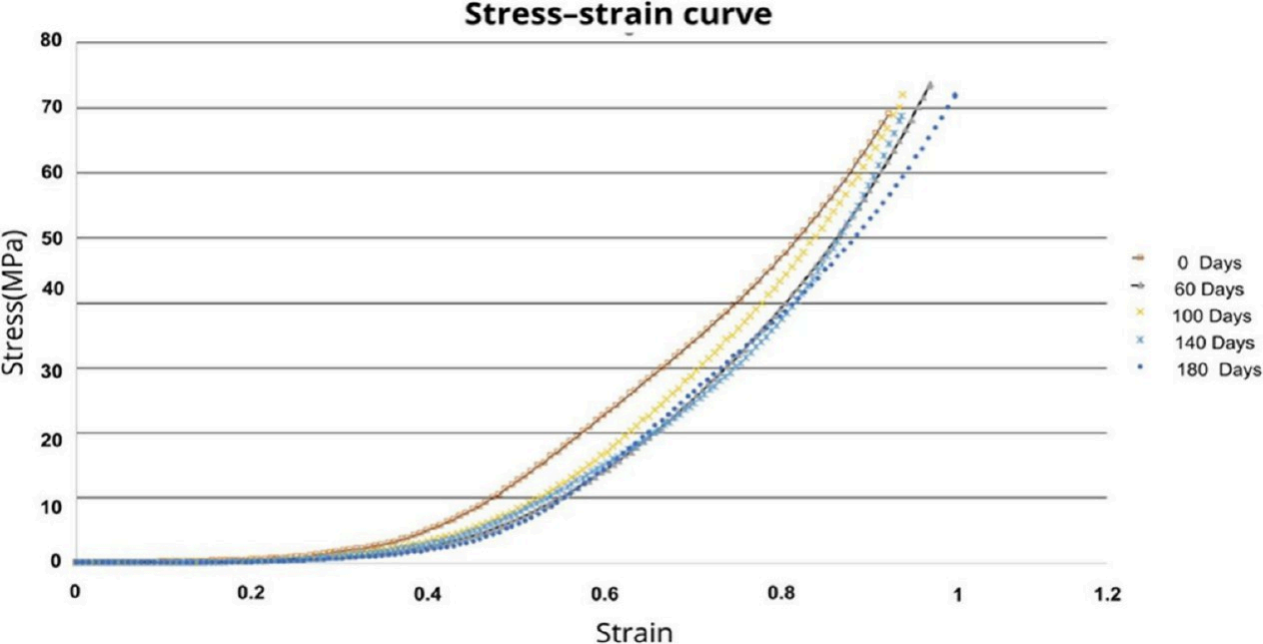
Stress–strain curves. The mechanical properties of scaffolds evaluated at different degradation time points.

The Young’s modulus was calculated for the scaffolds at different degradation time points (Figure 6A). The average values were 3.77 MPa on day 0, 4.57 MPa on day 60, 4.59 MPa on day 100, 5.46 MPa on day 140, and 3.67 MPa on day 180. No statistically significant differences were observed between the groups.

**Figure 6:**
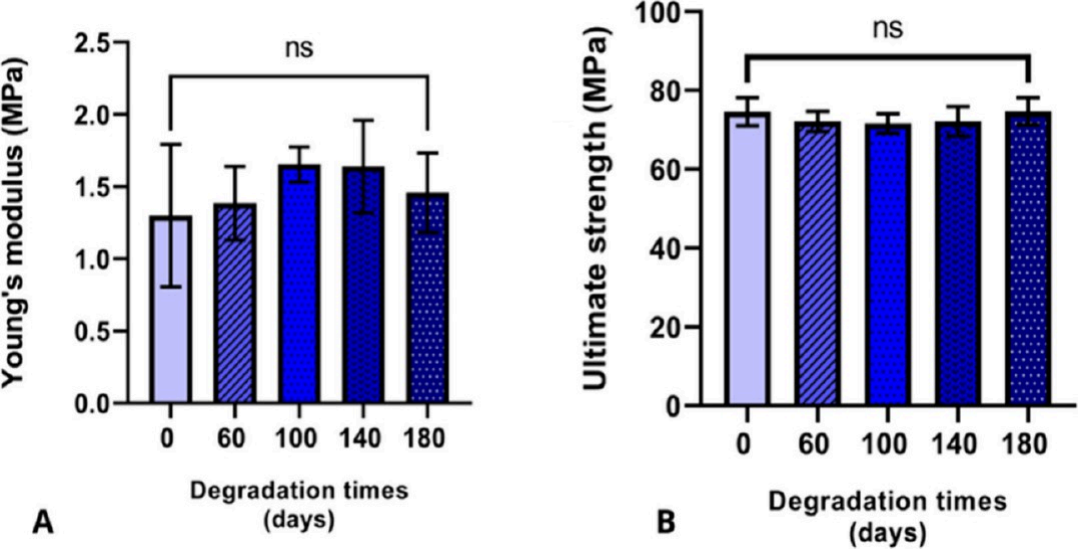
Young’s modulus and Ultimate strength. No statistically significant differences were observed between the groups in either Young’s modulus (A) or strength (B).

The average ultimate strength (Figure 6B) for each time point was as follows: 74.62 MPa (day 0), 72.18 MPa (day 60), 71.66 MPa (day 100), 72.2 MPa (day 140), and 74.71 MPa (day 180). No statistically significant differences were found between the groups.

### 3.5 In Vitro Assays with DPSC Cells

#### 3.5.1 WST-1 Cell Viability Assay

The WST-1 assay was performed on days 7 and 14 to evaluate cell viability. DPSCs were seeded onto scaffolds previously subjected to different degradation times. As shown in Figure 7, no statistically significant differences were observed between groups on days 1 and 7. However, significant differences were observed in all groups by day 14. These results align with expectations, as peak cell proliferation typically occurs during the initial days and subsequently declines as cells begin secreting extracellular matrix.

**Figure 7:**
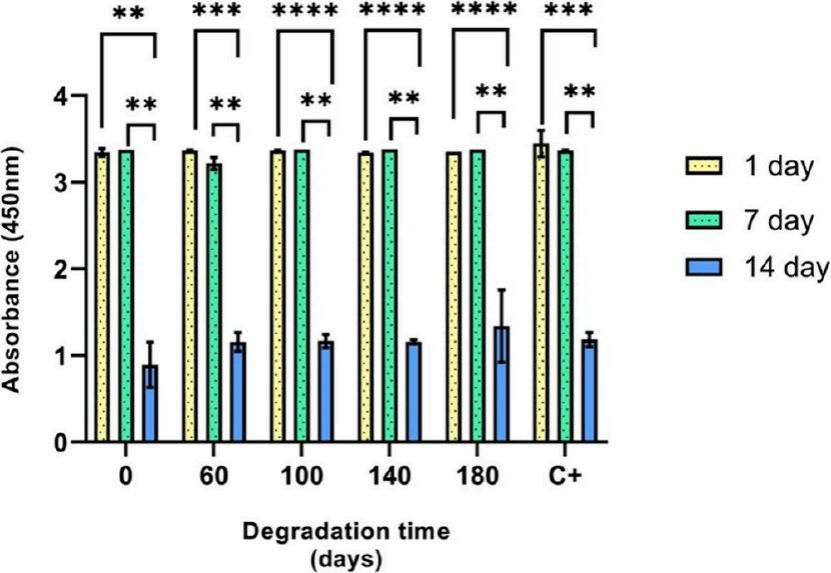
WST-1 assay on days 1, 7, and 14. The graph shows no statistically significant differences among the degradation groups on days 1 and 7; however, significant differences were observed across all groups on day 14. *p* < 0.01 (**), *p* < 0.001 (***), and *p* < 0.0001 (****).

#### 3.5.2 Alkaline Phosphatase (ALP) Assay

ALP assay was performed to evaluate the enzymatic activity of DPSCs cultured in osteogenic medium on days 7 and 14. As shown in Figure 8, the ALP activity was higher on day 7 than on day 14. Additionally, the only groups that did not show statistically significant differences between the scaffolds with different degradation times were those corresponding to days 60 and 100. Nevertheless, the scaffold degraded for 60 days was selected for in vivo implantation because its enzymatic activity did not decline sharply compared to the other groups.

**Figure 8:**
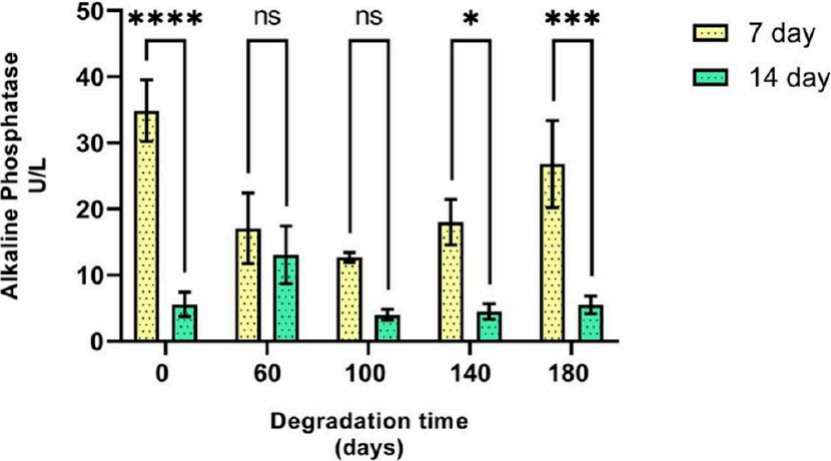
Alkaline phosphatase activity. The graph shows no statistically significant differences between the day 60 and day 100 groups. Notably, the scaffold degraded for 60 days and maintained a comparable ALP activity on days 7 and 14. *p* < 0.05 (*), *p* < 0.001 (***), and *p* < 0.0001 (****).

#### 3.5.3 Alizarin Red Staining (ARS) Assay and Quantification

Alizarin Red S (ARS) staining was performed on days 7 and 14 to evaluate calcium deposition on scaffolds subjected to different degradation periods. As shown in Figure 9, qualitative analysis revealed the formation of calcium nodules in all groups, primarily concentrated around the porous regions and between the printed filaments. An increase in staining intensity was observed with prolonged degradation, particularly on day 14, compared to the control group (scaffold without cells), suggesting enhanced secretion of mineralized extracellular matrix by the DPSCs.

**Figure 9:**
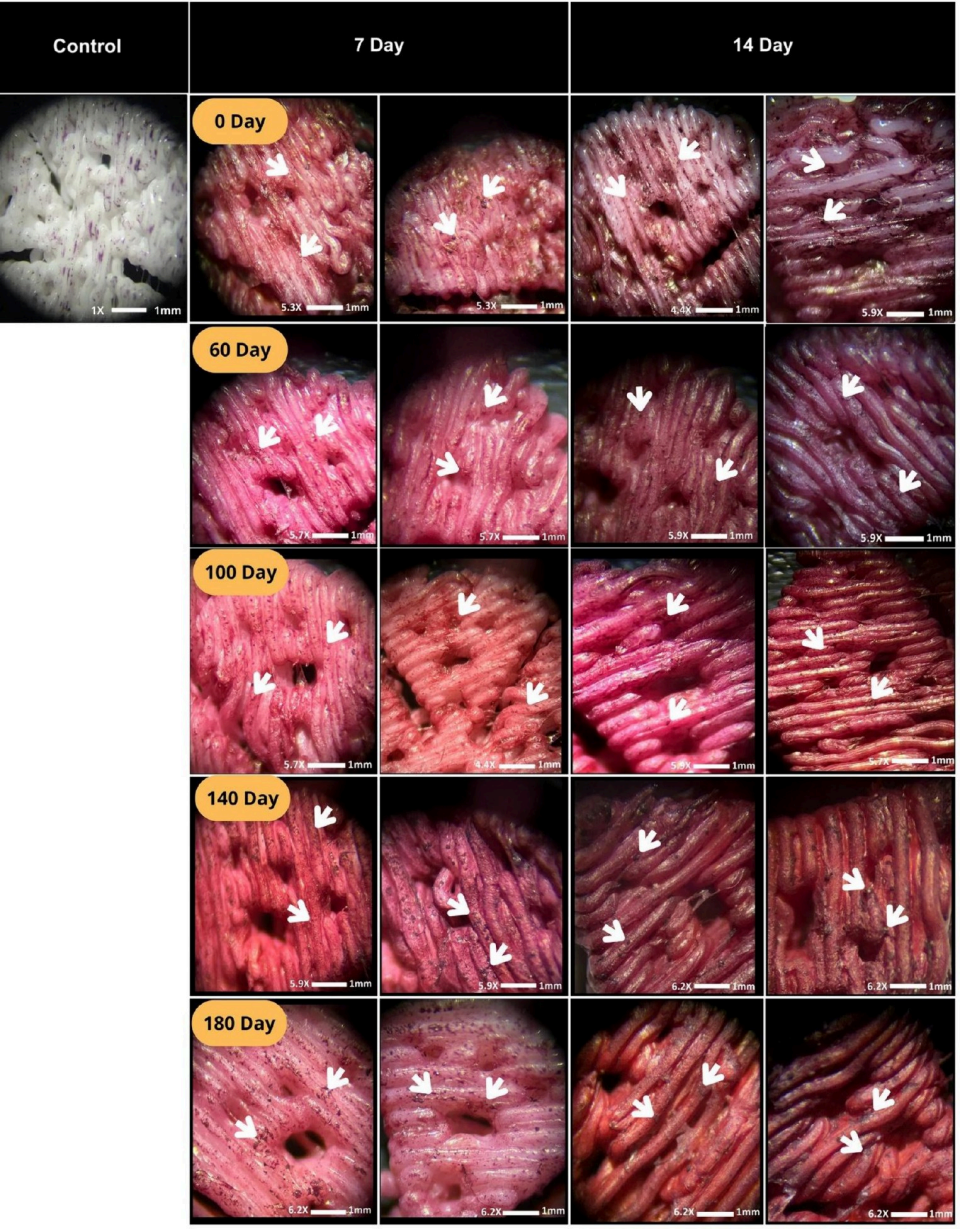
Representative images of scaffolds subjected to different degradation periods and stained with Alizarin Red. The left column shows staining performed on day 7, while the right column corresponds to day 14. The rows represent the scaffold degradation times (0, 60, 100, 140, 180 days, and control). Calcium deposits were observed on the scaffold surfaces in all experimental groups, regardless of degradation time. However, a greater staining intensity was evident in most scaffolds evaluated on day 14, which became more pronounced when compared to the control group.

Figure 10 shows a quantitative analysis of Alizarin Red extracted from the scaffolds. No statistically significant differences were found between days 7 and 14 or among the groups with different degradation times, except for the 60-day degradation group. This group showed a significantly higher ARS concentration on day 14 than on day 7.

**Figure 10:**
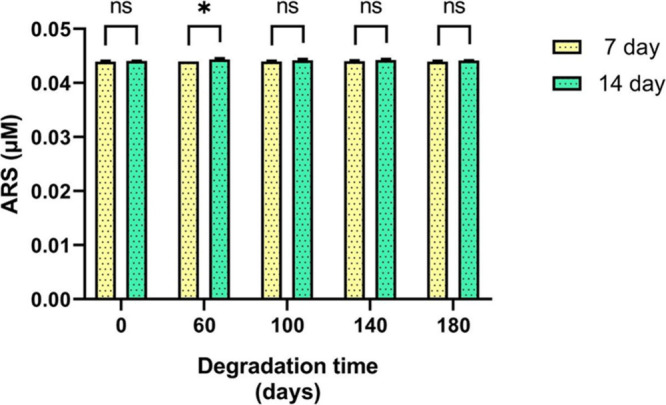
Quantification of Alizarin Red (μM) at days 7 and 14. The graph shows no statistically significant differences among the groups at different time points, except for the 60-day degradation group, in which day 14 exhibited a higher Alizarin Red concentration compared to day 7 (*p* < 0.05 (*)).

### 3.6 In Vivo Animal Model Assays

#### 3.6.1 Formation of Newly Formed Tissue within Bone Defects

Before the surgical procedures, the scaffolds were sterilized using hydrogen peroxide plasma. Critical-sized calvarial defects were created in 20 Wistar rats that were randomly assigned to four groups (n = 5 per group). Based on the in vitro cellular results, the 60-day predegraded scaffold was selected for use in Experimental Groups 1 and 3.

1) Control group: 3D-printed scaffold with graded porosity and three pore types (nondegraded).
2) Experimental Group 1:3D-printed scaffold (as described above), predegraded, and seeded with DPSCs in osteogenic medium (SDC).
3) Experimental Group 2:3D-printed scaffold (as described above), nondegraded, seeded with DPSCs in osteogenic medium (SWDC).
4) Experimental Group 3:3D-printed scaffold (as described above), predegraded without cells (SDWC).

Scaffolds were implanted into critical-sized calvarial defects according to the group assignments. Microcomputed tomography (μCT) was used to perform a descriptive evaluation at 30, 60, and 90 days postimplantation to assess newly formed bone tissue within the defect site.

Figure 11 shows a series of representative μCT images. The rows correspond to the experimental and control groups, and the columns represent the evaluation time points.

**Figure 11:**
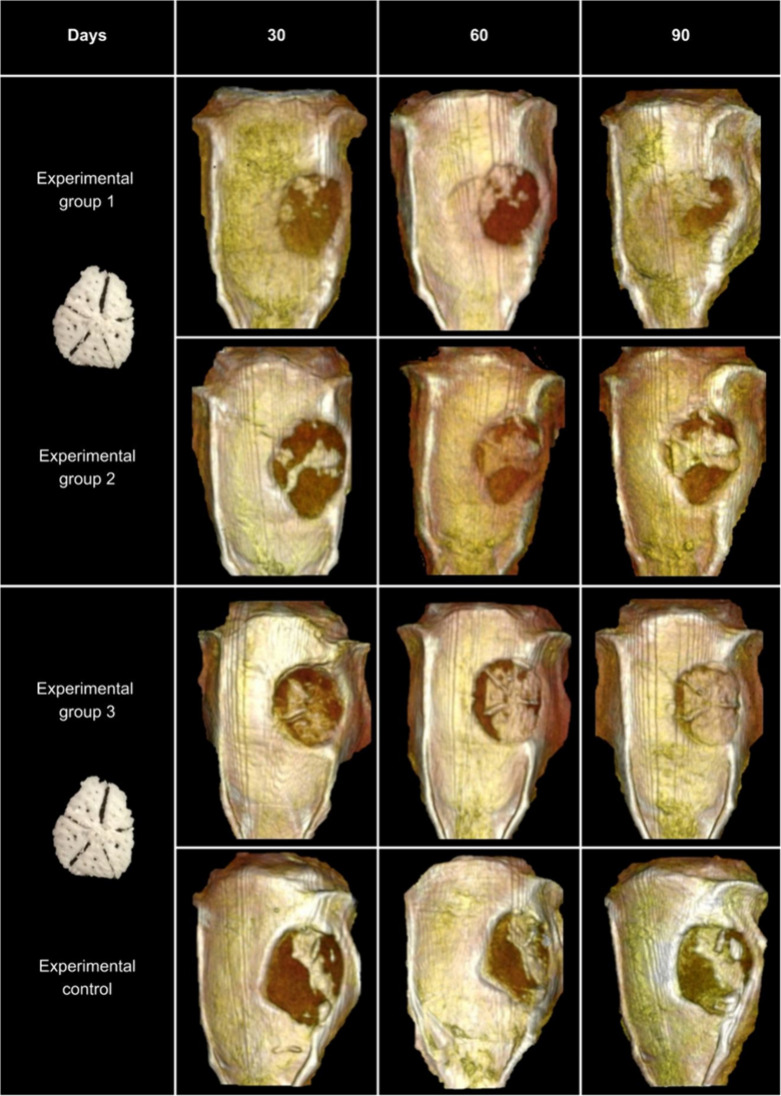
Mineralized new tissue formation. Representative images of newly formed mineralized tissues within the defect site. The rows correspond to the experimental and control groups, and the columns represent the different evaluation time points (30, 60, and 90 days).

In the control group, on day 30, newly formed tissue was observed spanning transversely across the defect, displaying a radiodensity comparable to that of the native bone. On days 60 and 90, the volume of the new tissue increased, extending both centrally and peripherally within the defect. In Experimental Group 1, a limited amount of new tissue was observed at the upper margin of the defect on day 30. By day 60, the tissue volume had increased and exhibited a radiodensity similar to that of the native bone. On day 90, new tissue covered approximately half of the defect, progressing from the periphery toward the center. In Experimental Group 2, on day 30, bridge-like tissue was visible within the defect, connecting the native bone to the defect center. On day 60, increased tissue formation and more areas of integration with the native bone were observed, effectively covering the center of the defect. A slight increase in tissue volume was noted on day 90 compared to day 60. In Experimental Group 3, on day 30, newly formed tissue was observed centrally within the defect, forming a bridge-like connection with the native bone. On day 60, greater tissue volumes and multiple integration points were observed, primarily in the regions formed by open pores or microchannels. These findings suggest that open pores play a key role in guiding tissue formation toward the center of the defect. By day 90, more than 50% of the defect had been filled with new tissue, exhibiting anatomical characteristics resembling native bone.

#### 3.6.2 Bone Mineral Density (BMD)

To confirm that the newly formed tissue observed in the micro-CT images was mineralized, the bone mineral density (BMD) of three distinct regions—native bone, scaffold, and newly formed tissue—was quantified.

The first group corresponded to the control group (Figure 12A). At 30 days, the BMD of the newly formed tissue (65.14 mg/cm^3^) was not significantly different from that of the scaffold (44.13 mg/cm^3^). However, by day 60, the BMD of the new tissue increased (70.88 mg/cm^3^), showing a statistically significant difference from that of the scaffold and native bone (106.94 mg/cm^3^). At day 90, the BMD of the newly formed tissue increased further (96.34 mg/cm^3^), showing a significant difference compared to the scaffold but no statistical difference from the native bone (112.53 mg/cm^3^).

**Figure 12:**
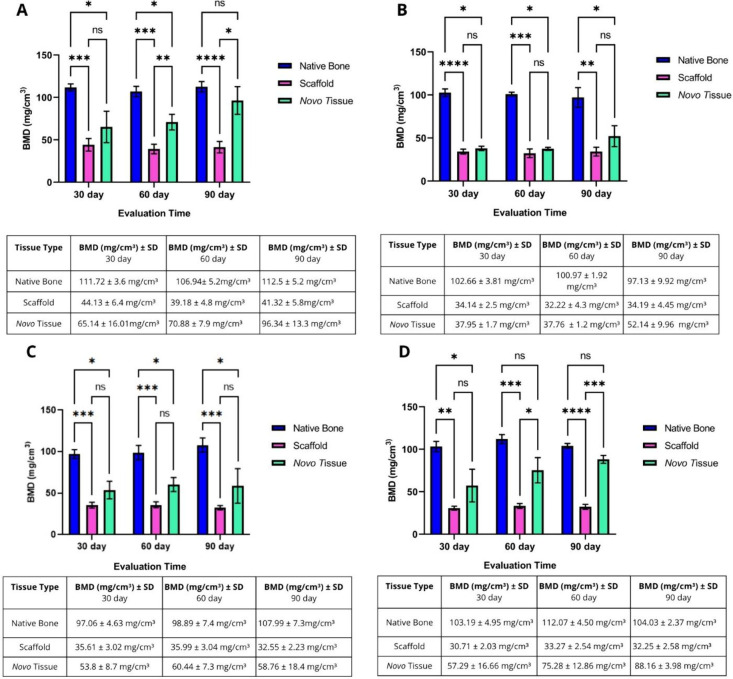
Bone mineral density (BMD). (A) BMD of the control group. The BMDs of the three tissues (native bone, scaffold, and newly formed tissue) were evaluated. The graph shows that by day 90, the BMD of the newly formed tissue was comparable to that of the native bone, with no statistically significant difference. (B) BMD of experimental group 1. The graph shows that by day 90, the newly formed tissue still exhibited a statistically significant difference in density compared with native bone. (C) BMD of experimental group 2. The graph shows that on day 90, the BMD of the newly formed tissue remained lower than that of the native bone, indicating a statistically significant difference. (D) BMD of experimental group 3. The graph demonstrates that from day 60 onward, the newly formed tissue exhibits a BMD comparable to that of the native bone, with a further increase observed on day 90. *p* < 0.05 (*), *p* < 0.01 (**), *p* < 0.001 (***), and *p* < 0.0001 (****). A complementary table shows the mean values and standard deviations of the BMD for each tissue evaluated in all experimental and control groups.

In experimental group 1 (Figure 12B), the newly formed tissue at 30 days exhibited a BMD of 37.95 mg/cm^3^, with no statistical difference compared to the scaffold (34.14 mg/cm^3^). On day 60, the BMD of the new tissue increased to 37.76 mg/cm^3^, showing a significant difference compared with the native bone.

To determine whether the newly formed tissue observed in the micro-CT images was mineralized, bone mineral density (BMD) was measured in three distinct regions: native bone, scaffold, and newly formed tissue.

In the control group (Figure 12A), the BMD of the newly formed tissue at 30 days (65.14 mg/cm^3^) was not significantly different from that of the scaffold (44.13 mg/cm^3^). However, by day 60, the BMD of the new tissue increased to 70.88 mg/cm^3^, showing significant differences compared to both the scaffold and native bone (106.94 mg/cm^3^). At day 90, the BMD of the newly formed tissue further increased to 96.34 mg/cm^3^, which remained significantly different from that of the scaffold but showed no statistical difference from that of the native bone (112.53 mg/cm^3^).

In experimental group 1 (Figure 12B), the newly formed tissue at 30 days had a BMD of 37.95 mg/cm^3^, with no significant difference from the scaffold (34.14 mg/cm^3^). On day 60, the BMD of the new tissue increased to 37.76 mg/cm^3^, showing a significant difference compared with native bone (100.97 mg/cm^3^). By day 90, the BMD had slightly increased to 52.14 mg/cm^3^ yet remained significantly different from the native bone (97.13 mg/cm^3^).

In experimental group 2 (Figure 12C), the newly formed tissue exhibited a BMD of 53.8 mg/cm^3^ at 30 days, with no significant difference from the scaffold (35.61 mg/cm^3^). On day 60, the BMD of the new tissue was 60.44 mg/cm^3^, which did not differ significantly from that of the scaffold. By day 90, the new tissue had a BMD of 58.76 mg/cm^3^, which remained significantly lower than native bone (107.99 mg/cm^3^), indicating persistent differences in mineral density.

In experimental group 3 (Figure 12D), the newly formed tissue exhibited a BMD of 57.29 mg/cm^3^ at 30 days, showing no statistically significant difference from the scaffold (30.71 mg/cm^3^). By day 60, the BMD of the new tissue had increased to 75.28 mg/cm^3^, resulting in no statistical difference from the native bone (112.02 mg/cm^3^). Furthermore, on day 90, the newly formed tissue reached a BMD of 88.16 mg/cm^3^, maintaining a density comparable to that of the native bone.

## 4 Discussion

Tissue engineering aims to restore or replace damaged biological structures by integrating scaffolds, cells, and signaling molecules. Central to this approach is the design of biomaterials that support cellular activity and integrate structurally and functionally with the host tissue.^49,50^ In the context of craniofacial regeneration, personalized scaffolds that match the geometry of the defect and provide optimized internal architecture are essential. In this study, we implemented a strategy to fabricate anatomically customized scaffolds with gradient porosity using Fused Deposition Modeling (FDM) based on medical imaging data to replicate the geometry of critical-sized cranial defects. FDM has already been applied in various medical fields, including orthopedics, craniofacial reconstruction, and personalized surgical guides, demonstrating its translational potential.^51,52^ Our approach aligns the structural features with the biological requirements for osteogenesis and vascularization.

The present study confirmed the feasibility of using FDM to fabricate scaffolds with gradient porosity tailored to critical-size calvarial defects in rats. Although FDM has resolution limitations, adjustments in printing parameters allowed us to achieve pore sizes aligned with osteoconductive requirements.^43,53–55^ Although FDM is one of the most accessible and cost-effective additive manufacturing (AM) methods, it shows limitations in resolution compared to other AM technologies.^56,57^ Nonetheless, the printed scaffolds were dimensionally accurate, supporting the capability of commercial FDM printers to produce geometrically complex constructs.

Achieving precision requires optimal adjustment of process parameters (nozzle temperature, print speed, infill density, and layer height), particularly given the use of polylactic acid (PLA), a polymer known for its dimensional instability due to shrinkage and residual stress. Some of these parameters match those described by Abas et al. and were crucial for maintaining pore geometry and interconnectivity.^58^

The scaffold design included three distinct pore types: closed, blind, and open (microchannels), which were strategically positioned to guide the biological response. These pores were previously reported by Chung et al.^59^ Microchannels oriented horizontally toward the native bone may allow communication with the periosteal layers, potentially enhancing osteoprogenitor cell recruitment and angiogenic support.^60,61^ The pore diameters, ranging from 234 μm at the periphery to 474 μm at the center, were within the range reported to favor osteogenesis and angiogenesis.^62–64^

A preimplantation hydrolytic degradation protocol was applied to accelerate the in vivo degradation of PLA. The scaffolds were immersed in Ringer’s solution at 37 °C for up to 180 days. The monitored parameters included surface morphology, pH change, mass loss, and mechanical integrity. SEM analysis revealed minimal surface erosion early, with more pronounced surface roughness after extended exposure. Although the weight loss was not linear or statistically significant, pH monitoring confirmed the ongoing hydrolytic activity through progressive alkalinization of the medium. This effect is attributed to the breakdown of PLA into lactic acid, which dissociates in buffered solutions, such as PBS or Ringer’s solution, to form lactate. In the presence of physiological cations (e.g., Na^+^, K^+^, and Ca^2+^), this process can lead to the formation of lactate salts, contributing to the observed pH increase.^65^

However, the lack of significant mass loss could be related to the high molecular weight of PLA (182,000 g/mol) and the relatively large size of the scaffolds compared with the microparticles described in other studies.^66–68^ Additionally, the surface changes observed via SEM were minimal during the early degradation stages, and the overall weight loss did not follow the expected trend. This result contrasts with the results reported by Spenlehauer et al.,^69^ who observed an 80% reduction in mass and distinct surface changes in PLA microspheres after 12 weeks of hydrolysis. The observed divergence in the degradation behavior may reflect the combined effects of the differences in polymer molecular weight and sample geometry.

Moreover, the increase in weight observed in most groups during the degradation period may be attributed to a phenomenon known as swelling, which involves fluid absorption by the scaffold. Prolonged exposure to aqueous media can promote fluid penetration into the biomaterial’s structure, particularly in areas with surface imperfections. This behavior has been documented as part of the typical response of PLA in physiological environments.^70,71^ Importantly, this apparent weight gain does not reflect an actual increase in polymer mass, but rather the accumulation of absorbed fluid.

Furthermore, the degradation process did not critically affect the mechanical integrity of the scaffolds, which maintained a stable elastic modulus and maximum stress during the degradation period. These findings support hydrolytically pretreated PLA scaffolds for *in vivo* implantation without compromising the structural support. Although in vitro degradation did not result in significant mass loss or mechanical weakening of the scaffolds, the 60-day predegraded group exhibited enhanced cellular responses and osteoconductive behavior. This suggests that hydrolytic treatment may induce subtle changes in surface roughness or surface chemistry,^72^ which may be sufficient to enhance protein adsorption and cell–material interactions.

The printed scaffolds exhibited an average porosity of 64.5%, comparable to that of trabecular bone and significantly higher than that of cortical bone, where the defect was located. Despite this mismatch, increased porosity can enhance nutrient diffusion and oxygen exchange, which is beneficial in the early regeneration stages.^73,74^ Mechanical testing showed a lower elastic modulus and ultimate stress than those of the native cortical bone, which was attributed to the scaffold architecture and porosity.^75^ However, given the minimal mechanical demands on the calvarial site, this is not considered a limiting factor for osteogenesis.

In vitro assays demonstrated the biocompatibility and osteogenic potential of dental pulp stem cells (DPSCs) seeded on 3D-printed scaffolds. Cell viability was maintained until day 14, after which a decline was observed. This finding is consistent with previous studies reporting peak cell proliferation during the early stages of culture.^76^

Alkaline phosphatase (ALP) activity increased on day 7 and remained stable through day 14. It is important to note that the decrease in ALP activity observed on day 14 does not necessarily indicate an inhibition of the osteogenic process; instead, it may be related to reduced cell proliferation as part of osteoblastic maturation and extracellular matrix (ECM) secretion. This interpretation is supported by the cell viability assay results, which showed a reduction in the cell population, likely associated with a functional shift in the behavior of cells adhered to the scaffold.

Similar trends have been reported by Waletzko et al. (2023), who observed a decrease in ALP activity on day 14 in mesenchymal stem cells (MSCs), including AD-MSCs and BM-MSCs, along with an increase in BGLAP (osteocalcin) expression, a late-stage marker of osteogenic differentiation.^77^ Other studies have also reported similar findings that support the physiological decline in ALP during later stages of osteogenic differentiation.^77–79^

Although the in vitro results suggest the onset of osteogenic differentiation, future studies should incorporate complementary molecular analyses such as real-time PCR to confirm the expression of lineage-specific genes.

On the other hand, Alizarin Red staining confirmed mineralized matrix formation, particularly concentrated at pore junctions and filament spaces, suggesting localized osteogenic differentiation.

Among the degradation groups, scaffolds predegraded for 60 days demonstrated the most favorable cellular responses and were selected for in vivo studies. This decision was based on the in vitro results, where this group exhibited higher calcium deposition at day 14, as determined by quantitative Alizarin Red S extraction.

Additionally, although alkaline phosphatase (ALP) activity decreased across all groups on day 14, the decline was less pronounced in the 60-day group. While cell viability also decreased in all groups at this time point, the relative preservation of osteogenic activity observed in the 60-day group supported its selection for the subsequent experimental phase.

In vivo, micro-CT analysis demonstrated clear evidence of new bone formation in all experimental and control groups. Remarkably, the most significant regeneration occurred in noncellular scaffolds with gradient porosity, both with and without predegradation. These results suggest that scaffold architecture alone can provide sufficient structural and biological cues to guide bone healing. The less effective performance of DPSCs-loaded scaffolds may be due to reduced cell survival postimplantation, poor initial vascularization, or inflammation-induced apoptosis. These findings align with prior observations that implanted mesenchymal stem cells may fail to survive or integrate if the host environment is not sufficiently supportive.^80–82^ Importantly, tissue mineral density in the best-performing groups reached levels that were statistically indistinguishable from native bone by day 90, confirming the presence and maturation of regenerated bone tissue.

This study contributes to the field of craniofacial tissue engineering by highlighting the process of converting tomographic images of critical-size bone defects into printable STL files, which constitutes a key aspect of this manuscript. It demonstrates that scaffolds with patient-specific geometry and graded porosity can be fabricated using accessible, low-cost FDM technology. Unlike other studies that rely on complex or high-resolution bioprinting platforms, our approach employs commercially available equipment to achieve precise control over pore architecture and anatomical conformity, offering a practical and scalable strategy for scaffold fabrication in regenerative medicine.^83,84^ Our findings underscore the potential of these architecturally optimized FDM-printed scaffolds to support bone regeneration in critical-sized calvarial defects, even in the absence of cellular supplementation. Notably, both predegraded and nondegraded acellular scaffolds elicited more favorable osteogenic responses than their cell-seeded counterparts, with evidence of new bone formation extending to the center of the defect. This outcome suggests that the scaffold surface features alone were sufficient to promote osteoconduction, highlighting their translational potential in clinical applications. Nonetheless, this study had certain limitations. Histological analyses to evaluate tissue organization and molecular assays to confirm osteogenic differentiation were not performed, and the long-term mechanical performance of the scaffold postimplantation remains uncharacterized. These aspects warrant further investigation to validate the efficacy and clinical utility of the scaffolds fully.

## 5 Conclusions

This study demonstrates that anatomically customized 3D-printed PLA scaffolds with gradient porosity fabricated using Fused Deposition Modeling (FDM) can maintain their surface and mechanical integrity following in vitro hydrolytic degradation. Although no significant structural changes were observed, cell-based assays revealed consistent viability across all degradation periods, with enhanced osteogenic differentiation at day 60, indicating a biologically favorable pretreatment window. Further molecular analysis will be required to confirm lineage-specific differentiation. *In vivo*, micro-CT analysis confirmed the formation of new bone tissue within the center of critical-size calvarial defects, an area typically challenging to regenerate. Notably, acellular scaffolds achieved bone mineral density values comparable to native bone, underscoring the role of scaffold architecture alone in promoting osteogenesis. These findings support the translational potential of personalized, FDM-fabricated scaffolds as a viable strategy for craniofacial bone tissue engineering.
